# Machine Learning Spectroscopy Using a 2-Stage, Generalized Constituent Contribution Protocol

**DOI:** 10.34133/research.0115

**Published:** 2023-04-20

**Authors:** Jinming Fan, Chao Qian, Shaodong Zhou

**Affiliations:** ^1^ College of Chemical and Biological Engineering, Zhejiang Provincial Key Laboratory of Advanced Chemical Engineering Manufacture Technology, Zhejiang University, 310027 Hangzhou, P. R. China.; ^2^ Institute of Zhejiang University - Quzhou, Zheda Rd. #99, 324000 Quzhou, P. R. China.

## Abstract

A corrected group contribution (CGC)–molecule contribution (MC)–Bayesian neural network (BNN) protocol for accurate prediction of absorption spectra is presented. Upon combination of BNN with CGC methods, the full absorption spectra of various molecules are afforded accurately and efficiently—by using only a small dataset for training. Here, with a small training sample (<100), accurate prediction of maximum wavelength for single molecules is afforded with the first stage of the protocol; by contrast, previously reported machine learning (ML) methods require >1,000 samples to ensure the accuracy of prediction. Furthermore, with <500 samples, the mean square error in the prediction of full ultraviolet spectra reaches <2%; for comparison, ML models with molecular SMILES for training require a much larger dataset (>2,000) to achieve comparable accuracy. Moreover, by employing an MC method designed specifically for CGC that properly interprets the mixing rule, the spectra of mixtures are obtained with high accuracy. The logical origins of the good performance of the protocol are discussed in detail. Considering that such a constituent contribution protocol combines chemical principles and data-driven tools, most likely, it will be proven efficient to solve molecular-property-relevant problems in wider fields.

## Introduction

The current rapid development of the chemical and material industry requires quick and accurate prediction of various properties, which is, however, challenged by the limited efficiency of quantum mechanical computing [1]. Coming into the era of big data, nowadays, data-driven machine learning (ML) [2,3] has been widely used in the fields of materials [4–7] and chemistry [8,9]; a series of molecular properties can thus be provided with trained models using enough samples [6,10,11], which has greatly accelerated the discovery of new functional molecules [12]. In the prediction of chemical properties, it is necessary to transform the chemical structure into computer-readable forms. To this end, molecular descriptors [13] and molecular fingerprints [14] have proven to be universal for the prediction of various properties. However, these characteristics may not directly correlate with the nature of molecules as large datasets are necessary to achieve satisfying accuracy. Therefore, when expanding ML prediction into new areas, insufficient experimental data limit the feasibility—in spite of the state-of-the-art algorithms, and one can only rely on quantum mechanical calculation [15]. The relatively low computing efficiency and high cost impede high-throughput analyses [16]. Recently, it has been reported that ML in combination with quantum chemical calculation using quantum descriptors may reduce the computation cost and afford good results [17]. However, this strategy is still difficult for small samples. Moreover, due to the lack of data for mixtures, both the data-driven ML models and quantum chemical calculations are less efficient to predict the properties of mixtures. Therefore, predicting the properties of mixtures with a limited amount of data remains challenging for ML-based protocols.

The prediction of spectral properties often depends on computer modeling. For example, time dependent-density functional theory (TD-DFT) has been employed to predict the electron absorption spectrum and maximum absorption wavelength [18–20]. In fact, TD-DFT has been developed for decades and achieved success in spectra prediction [21]. Recently, quite a few ML prediction methods have been employed based on molecular descriptors and molecular fingerprints [22–25], while these methods are adapted to a specific sort of dyes. This may result from both the lack of large databases of data and the insufficient rationality of the employed descriptors. In addition, ML methods have also been combined with quantum chemical calculations so as to accelerate the consuming computation of spectral data [26]. On the other hand, even if the data for dye mixtures were sufficient, the above models are not adapted to the prediction of mixed spectra.

Group contribution (GC) is a classic method that has been traditionally used in predicting thermodynamic properties [27–29]. However, the cost and difficulty of its manual modeling limit its development in complex spectral properties. Driven by big data, deep learning transforms the representations of sample features in the original space into a new one through feature transformation stage by stage, which makes classification or prediction easier [30]. However, this is only due to data accumulation, ignoring the laws of nature. Therefore, elaborate combination of GC method and ML model is, most likely, able to realize the nature of the features and model them in a more rational manner. However, the traditional GC method [27–29] only describes the molecular composition at exactly the atomic level, while neither the configurational characteristics nor the electronic features can be directly expressed.

**Fig. 1.**
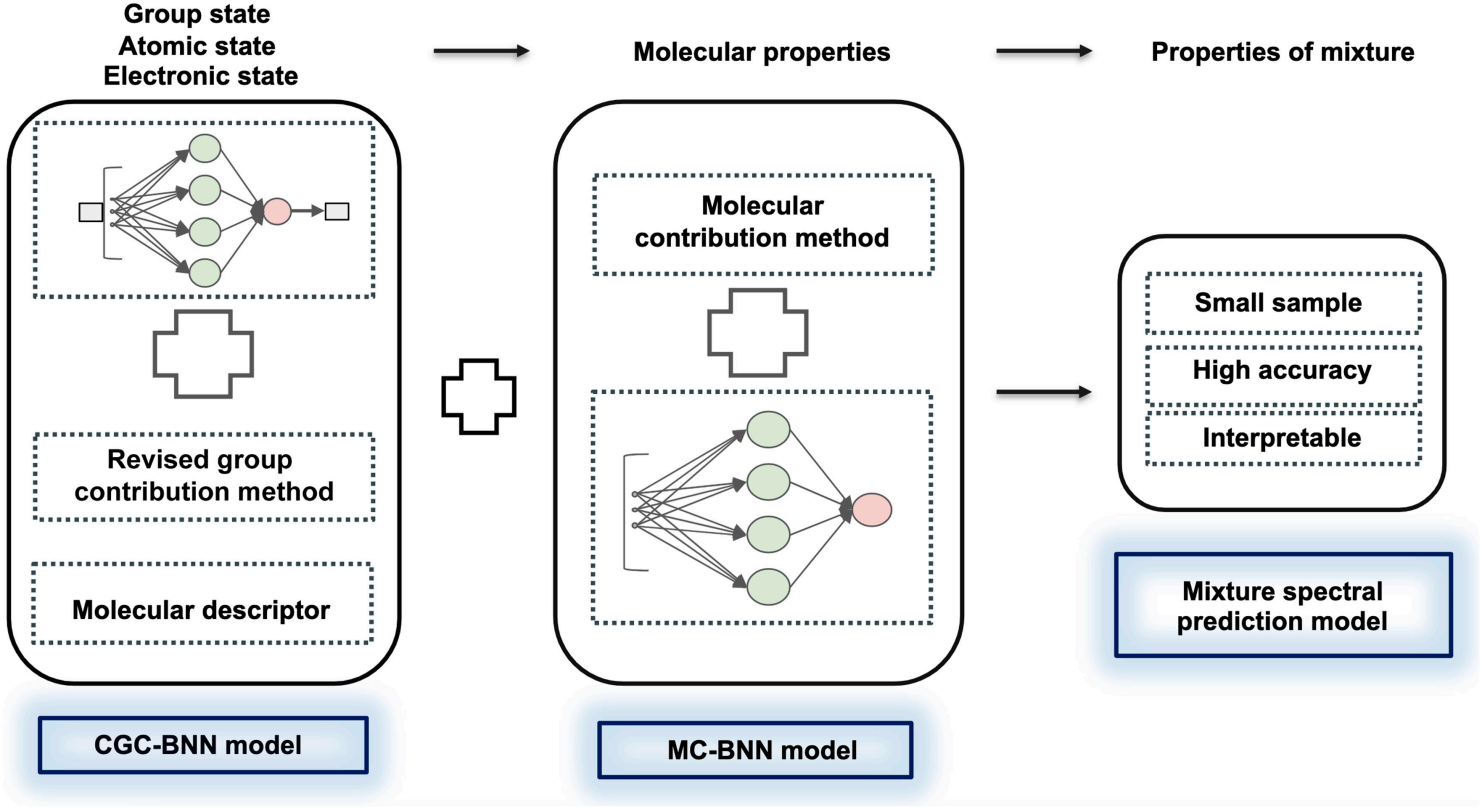
Flowchart of the prediction protocol as developed in this work.

Most of the current ML models use complex network models to fit big data, but such models are poor to be explained using principles of physical science. Thus, one can only rely on sufficient data so as to ensure the reliability of results [30]. Inspired by the GC method and deep learning, we combined their advantages and designed a 2-stage framework for the prediction of complex mixture properties; here, 2 independent neural network frameworks were employed in tandem (for a schematic illustration, see Fig. 1). In the first stage, the model employs neural network to predict the spectroscopic property of the molecule based on the self-revised GC (revGC) method, which is supplemented by descriptors concerning the electronic and atomic states. Here, we call it corrected group contribution (CGC) method. The second stage is another neural network model based on the molecule contribution (MC) method. The MC method is used to predict the properties of mixtures from the intermolecular state. To be more detailed, the MC method assumes that the properties of the mixture are due to the contributions of each molecule in a certain manner, and the ML model learns the superposition of each contribution (i.e., the mixing rule) to eventually output the properties of the mixture. The latter is normally expressed by complex intergroup interaction terms in traditional GC methods. Obviously, the 2-stage model designed here is explainable. Starting from the very elemental components of molecules and considering various factors influencing the properties from electrons to mixtures, each part of the framework can be formulated with chemical meaning while parameterized with ML. The more logical learning processes may greatly reduce the uncertainty of ML and avoid overfit. Moreover, such a framework should not require heavy load of calculation using huge data but need only a few samples, which include enough kinds of chemical groups.

Thus, the first stage of the model is capable of accurate prediction of maximum wavelength and full spectrum for single molecules with a much smaller training sample compared to the previously reported ML models. Furthermore, with the second stage of the model, the spectra for a multicomponent system can be obtained by training with only 2-component spectral data. More details are discussed as follows.

## Results and Discussion

### Prediction of full absorption spectra

As mentioned above, nowadays, data are becoming a sort of extremely valuable and scarce resource. Due to the lack of complete ultraviolet (UV)-visible absorption spectra and spectral data for mixtures, we cannot fully verify the performance of the entire 2-stage framework. In order to prove the superiority of our model, we will use as few data as possible to verify the 2 models logically.

**Fig. 2.**
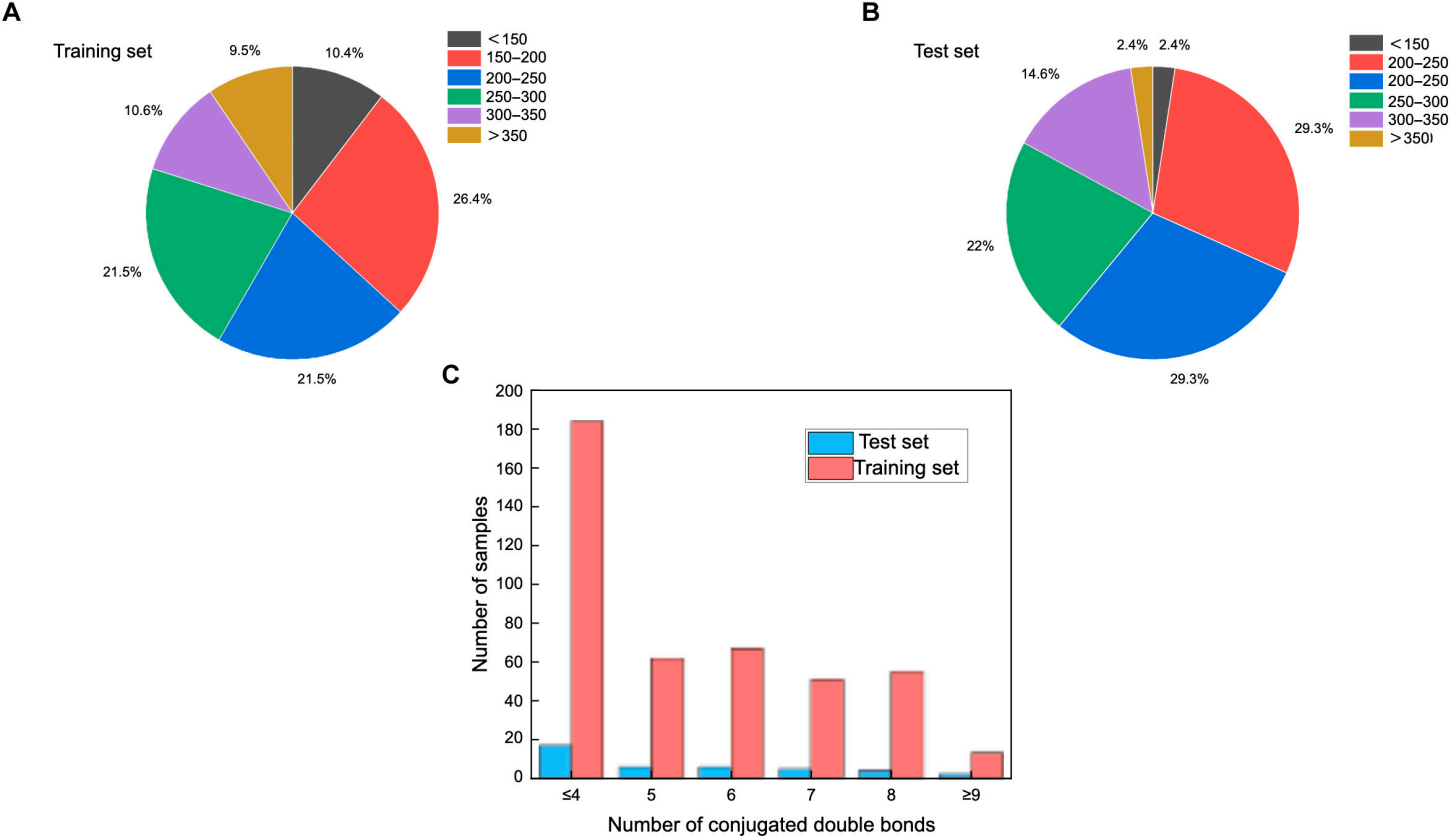
Sample features for full spectra prediction. (A) Masses of the molecules employed in the training set. (B) Masses of the molecules employed in the test set. (C) Distribution of the number of conjugated double bonds (the determination method of conjugated double bonds can be found in the Materials and Methods section) in the test and training sets.

**Fig. 3.**
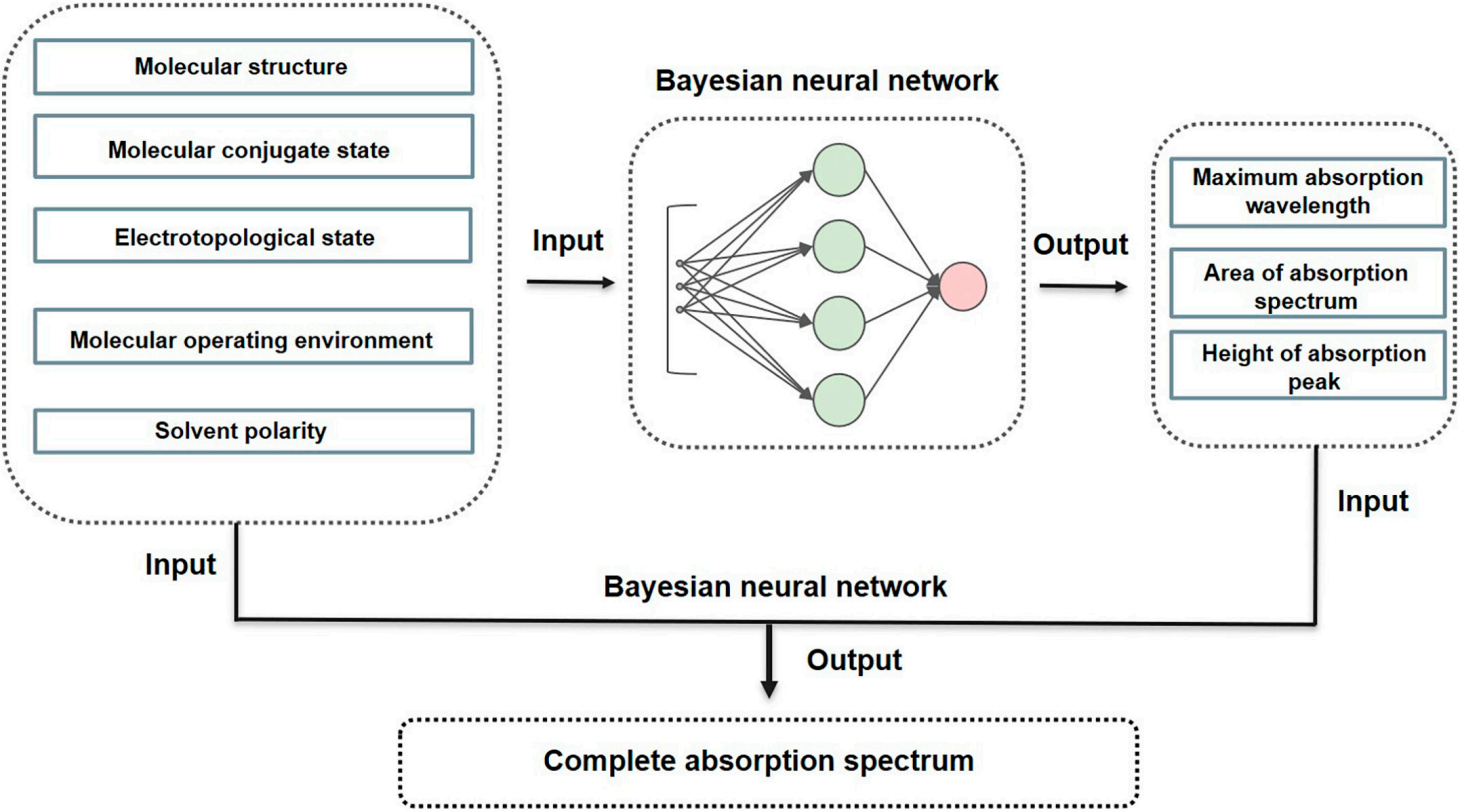
Structure of the CGC-BNN model. This model consists of 2 Bayesian neural networks (BNNs) in series. The first model uses the molecular composition features like the group contribution method, conjugation descriptor, electronic state descriptor, etc. as input, and outputs the maximum absorption wavelength, peak area, and absorption intensity; these results are employed next to correct the composition features to eventually afford the complete absorption spectrum with BNN.

First, we collected 432 groups of UV absorption spectra data from the literature as the training set (sample features are shown in Fig. 2; for more details, see the Materials and Methods section) and 40 groups of data as the test set to verify the performance of CGC-BNN model; the molecules in the dataset contain sufficient chemical groups (the name and chemical formula of the molecule in the dataset are provided in the Supplementary Materials). The mean absolute error (MAE), mean square error (MSE), and the coefficient of determination (*R*^2^) of the prediction results are presented in Table 1. According to our design, we only need to know the chemical formula of the molecule and convert it into a vector of features at the electronic, atomic, and group levels with the revised GC method and molecular descriptor (see the Materials and Methods section for more details); the vector form is used next as input to obtain the complete absorption spectrum (see Fig. 3 for the prediction process). Since we only use small samples, it is important to verify whether the models are overfitted in training. Thus, we randomly select 10% of the samples for internal prediction each time during the internal validation and repeat 50 times of prediction to afford an average value as the final result (see Table 1). With this method, each molecule in the dataset gets its turn for prediction. Next, we used the 432 sets of data for training to perform external validation using 40 molecules. As shown in Table 1, the error of external test is only slightly larger than that of internal verification. Moreover, due to the lack of vis-spectra data, the maximum absorption wavelengths in our training data may not be accurate. In fact, many of these molecules also have absorption peaks in the visible light band, which is supposed to reduce the accuracy of the model considerably. To further support our conclusion, we used another 73 groups of visible spectra data (this training set contains different dyes dissolved in different solvents; see the Materials and Methods section for details) for internal 10% validation, and the results are shown in Table 2 (the average values of 50 time repeats are presented here). As (somewhat) expected, due to the lack of full-spectrum data, although we used 432 sets of data for training, the prediction of UV spectra (which may not include the maximum absorption wavelengths of the molecules) is still unsatisfactory. By contrast, the small-sample training affords higher accuracy in the prediction of vis-spectra as compared to the training using 432 groups of UV-spectra data. Here, for comparison, Shao et al. [22] used ~1,200 samples in training to eventually obtain 1.52% mean relative error (MRE) in the prediction of maximum absorption wavelength with a self-designed ML system, SMFluo1-DP.

**Table 1.** Performance of the CGC-BNN model in the prediction of UV spectra.

Method	MAE	MSE	*R* ^2^
Training set	0.0783	0.0147	0.7782
Test set	0.1005	0.0227	0.6558

**Table 2.** Internal test results of the prediction of maximum absorption wavelength.

Method	MAE	MRE	*R* ^2^
Visible light spectrum	18.5383	0.0345	0.9183
Ultraviolet spectrum	17.0357	0.0612	0.1287

**Fig. 4.**
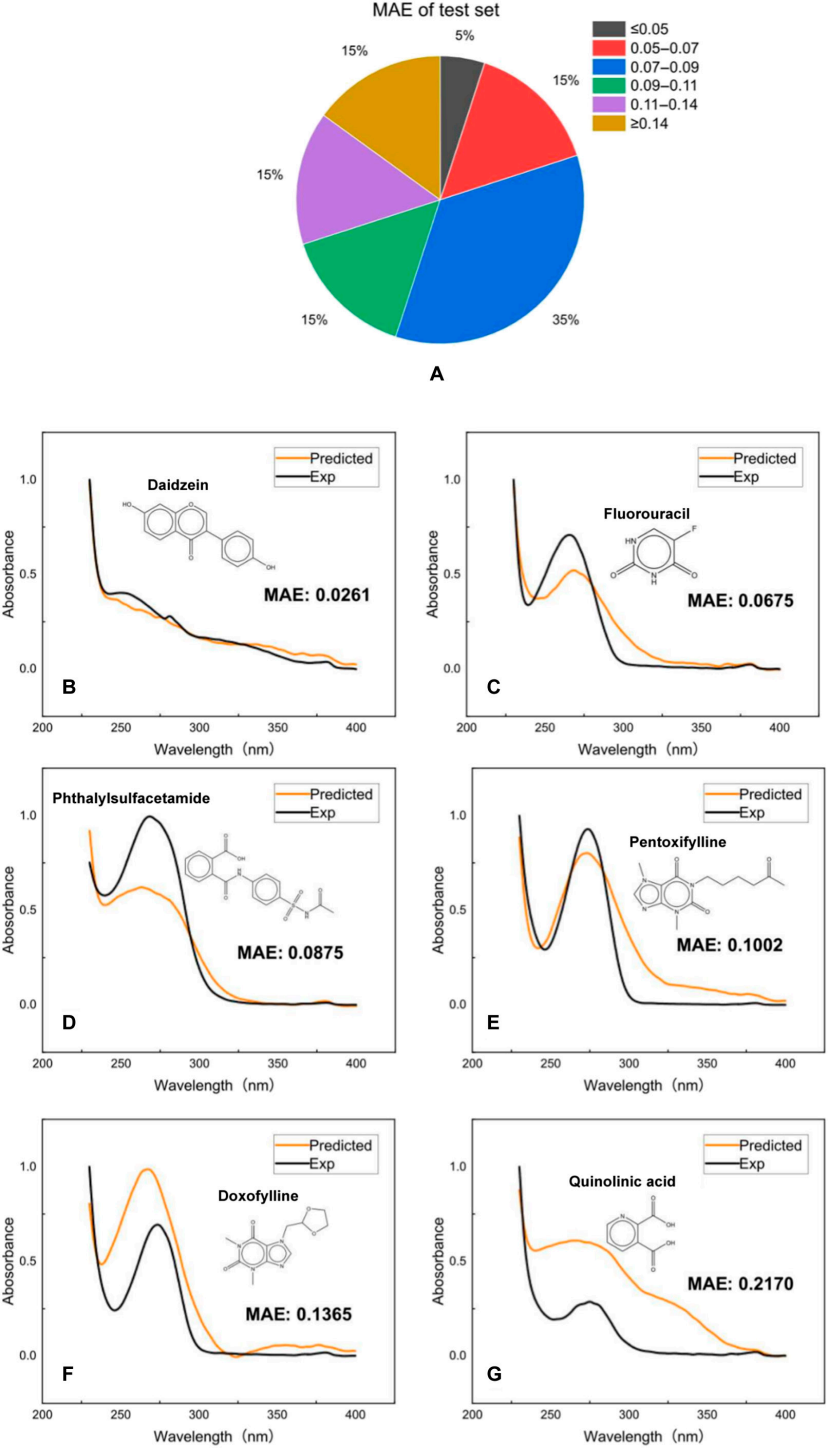
Error distribution for the 40 test samples (A). Examples of prediction and experimental spectra for (B) daidzein, (C) fluorouracil, (D) phthalylsulfacetamide, (E) pentoxifylline, (F) doxofylline, and (G) quinolinic acid.

Although the lack of data will dramatically reduce the accuracy of the ML model, the final prediction results are still excellent in our case. As shown in Fig. 4, there is no serious deviation between the predicted spectral waveform and the experimental value. Note that Urbina et al. [23] used more than 2,000 samples as the dataset but still could not converge successfully with the feedforward neural network. Zhang et al. [31] used quantitative calculation data as input and also used more than 2,000 sets of data to accurately predict the UV absorption spectrum of protein. To our delight, the CGC-Bayesian neural network (BNN) model in this work reaches comparable accuracy with the above 2 literatures but requires a much smaller dataset and much shorter time.

Compared with a data-driven model, the CGC model is more like a data-fitted chemical equation. Thus, a series of factors influential to the molecular properties have been taken into account to afford the accurate spectrum. This enables our model to adapt to small data samples. To demonstrate the crucial roles played by different descriptors in the model, we tried the revGC method and the molecular descriptor method (by describing the characteristics of atoms and charges) separately. As shown in Table 3, their performance is not as good as that of the CGC method. In addition, guided by chemical principles, the algorithm itself is not expected to dominate the accuracy of the prediction. To justify this, we also modeled the CGC method using other ML models. As a result, all predictions afford similar results (see Table S3 and Fig. S1).

### Prediction of mixed absorption spectra

**Fig. 5.**
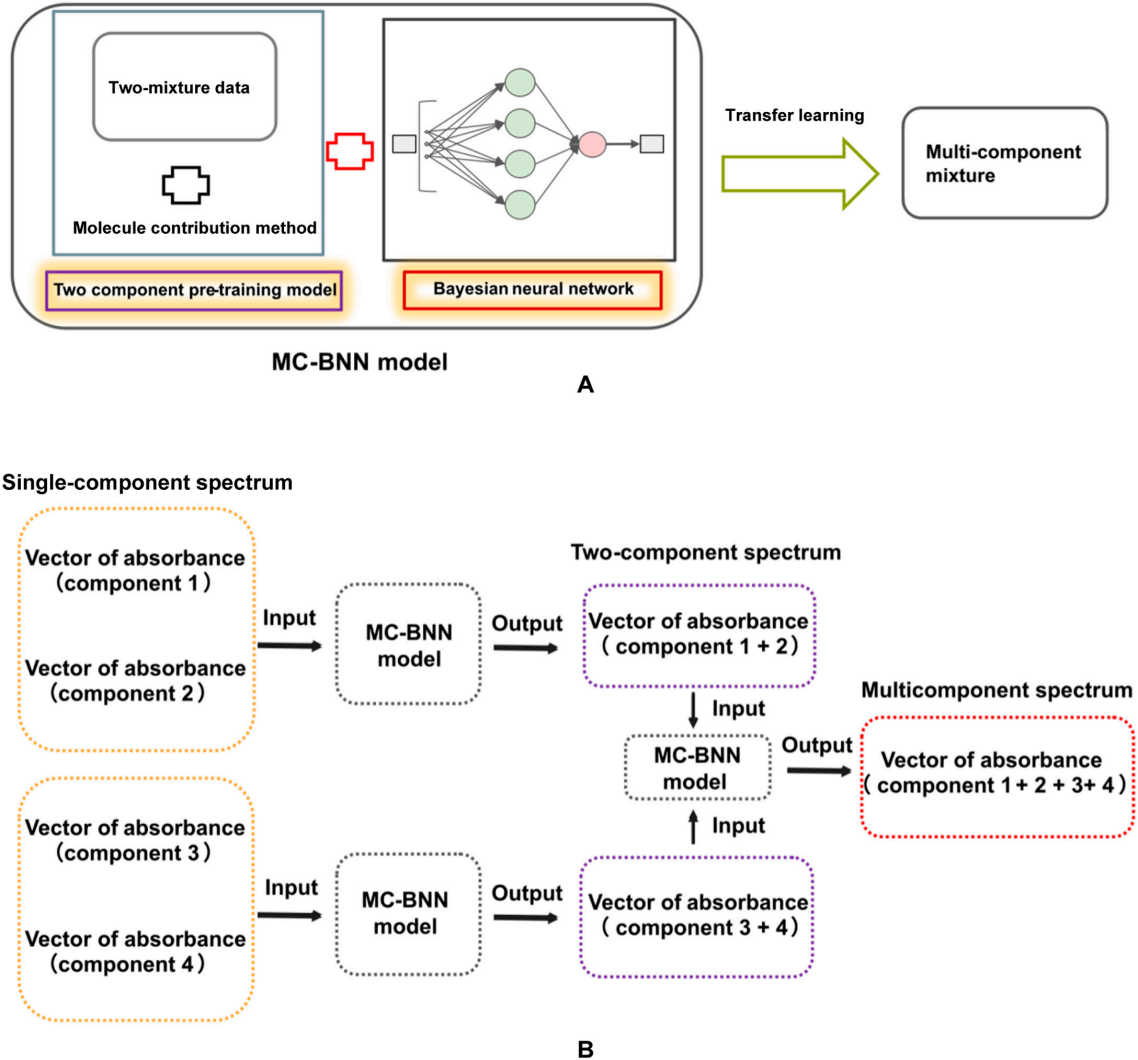
Flowchart of the MC-BNN prediction model as developed in this work. (A) Principle of the MC-BNN model. (B) The specific workflow of MC-BNN in predicting the mixing spectra of 2 components and the migration to multicomponent spectra.

After obtaining the absorbance vector of the complete absorption spectrum of a single molecule, it remains challenging to use these data to afford the spectra of mixture. To this end, we developed the MC method. Different from the GC method, the MC method determines the characteristics of the mixture based on the size of the characteristic value contributed by each molecule. It is constructed based on 2 assumptions: (a) the interactions between different molecules can be expressed as the mixing functions of group vectors (especially the difference between vectors); (b) the mixture properties are contributed by linear or nonlinear combination of each species upon considering both the mixing functions and the relative concentrations of different molecules. For the traditional GC method, the interactions of different groups in the same or different molecules are considered by introducing complex reciprocal parameters. In this work, after dealing with intramolecular group interactions in the first stage, the second stage treats intermolecular group interactions as functions of the group vector difference. According to the Beer–Lambert–Bouguer law, the absorption spectrum of the mixture is additive combination of the spectra of individual components. However, for a multicomponent system, the intermolecular interaction force will make the mixed spectrum show a nonlinear addition (see Fig. S2). Here, if one considers the intermolecular interaction by counting the contribution of each type of force, e.g., by employing energy decomposition analysis (EDA) [32–34], the entire model can be much too complex and may encounter convergence problems. Thus, we can count the interactions of different molecules by introducing a mixing function, as, for example, the following interaction factor *k_ij_* (Eq. 1):

$$k_{\mathrm{ij}}=A\left({\vec{a}}_{i}-{\vec{a}}_{j}\right)+B{\left({\vec{a}}_{i}-{\vec{a}}_{j}\right)}^{2}+C{\left({\vec{a}}_{i}-{\vec{a}}_{j}\right)}^{3}+\cdots$$ (1)

where ${\vec{a}}_{i}$ and ${\vec{a}}_{j}$ are the group vectors for the components *i* and *j*, and the spectrum for mixture can be obtained using Eq. 2:

$$L=\sum_{i=1}^{n}\left[L_{i}x_{i}+\sum_{i}^{n}\left(\frac{x_{i}x_{j}}{x_{i}+x_{j}}{\mathrm{Xk}}_{\mathrm{ij}}\right)\right]$$ (2)

in which *L_i_* is the absorption spectrum of pure component *i*, and *X* is the transformation vector. The logic represented by the above formula is the fundamental framework for the MC method, but it would be rather difficult for artificial modeling to provide relatively an accurate solution. Thanks to the data-driven tools, both Eqs. 1 and 2 can be modeled with the neural network during training with the 2-component data; we assume that the multicomponent interaction can be interpreted using the 2-component data in a functional manner afforded by selected neural network logics. Thus, the task left is to migrate the 2-component models to multicomponent systems without additional training of experimental data; such a transfer learning process is illustrated in Fig. 5. Notably, the contribution of solvents to the spectra via various solvent effects has been taken into account within the CGC model in the first stage. Therefore, one does not need to consider the influence of solvent in the MC-BNN model of the second stage.

**Fig. 6.**
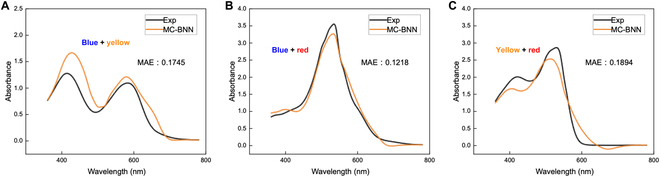
Prediction of spectra for 2-component systems. (A) Blue and yellow dyes. (B) Blue and red dyes. (C) Yellow and red dyes.

Up until here, we have been able to output the absorbance at each wavelength through the molecular formula. If the vector composed of this absorbance is put into the MC-BNN model, the spectrum of the multicomponent system can be obtained. There is no doubt that if a certain amount of complete UV-vis absorption spectra and the associated mixture spectra data were available, the 2-stage models could have predicted the mixture spectra with high accuracy. Unfortunately, such experimental data are not abundantly available in the literature. However, we can still verify the performance of the MC-BNN model separately. Here, we use only 73 groups of 2-component data as the training set. The MAE of internal test is 0.1576; *R*^2^ is 0.9496 (again, 10% of the samples were selected for test each time, and 50 repeats of prediction gave the average values as the final result). Then, we selected 3 dyes covering 3 primary colors and used the above 73 groups of data as the training set for prediction purposes. As shown in Fig. 6, the MC method has been proven from theory to practice—the MC-BNN model can learn the intermolecular interactions and output a relatively accurate spectrum for mixture. Considering the satisfactory performances of the first and second stages in modeling the intra- and intermolecular interactions, in principle, it is able to afford spectra of any organic systems upon training with sufficient data. Such sufficient data are expected to be much smaller than that required for traditional data-driven ML protocols.

**Fig. 7.**
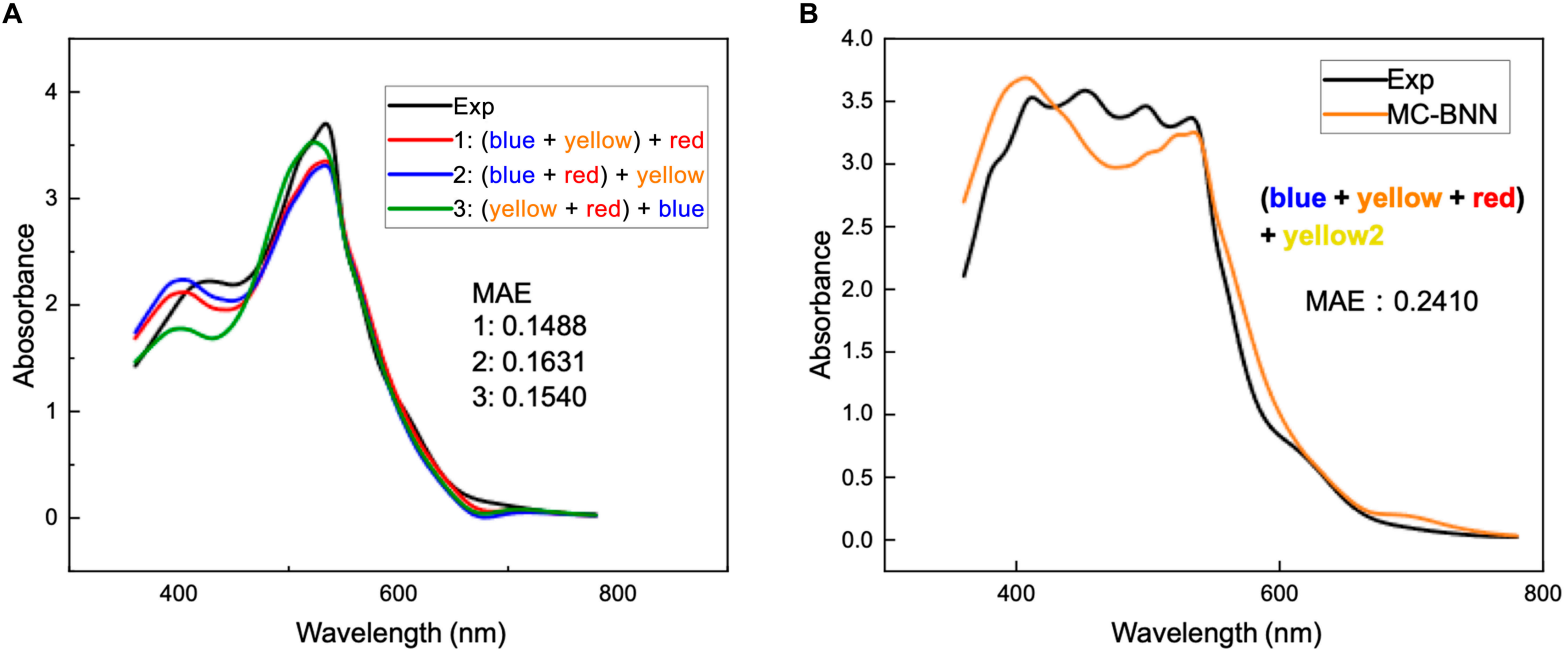
Prediction of spectra for multicomponent systems. (A) Three-component spectra obtained by different prediction methods. (B) Prediction results of 4-component spectra.

Finally, we extended the prediction of mixture spectra from a binary system to multicomponent systems. In order to compare the impact of different virtual mixing paths in prediction, we used 3 mixing paths to predict. Surprisingly and as expected, although multicomponent experimental data were not involved in training, the migration of the model to the ternary system did not enlarge the prediction error. As shown in Fig. 7, the prediction of multicomponent systems is satisfactory, and the error only comes from the relative intensity of absorbance. This not only proves the reliability of the MC-BNN model but also reflects its good migration ability and obvious modularity.

For the multicomponent mixture, the spectrum can be obtained accurately by using only 2-component data for training. Moreover, note that the MC method is logically available as well for other mixture properties: as long as the intermolecular interaction matters for the mixture properties, the 2-stage protocol may be operative.

## Conclusion

In summary, we have presented a 2-stage protocol for accurate prediction of absorption spectra for both single molecules and multicomponent mixtures. The architecture of the protocol is based on the CGC method and a new MC method as developed in this work; the BNN model is employed for data processing of each stage. In the prediction of full absorption spectra, the CGC-BNN model can provide high accuracy while using a rather small dataset for training. By contrast, previously reported ML protocols require much more samples to ensure the accuracy of prediction. Furthermore, by incorporating the MC method with mixing rules as modeled by BNN, the absorption spectra for multicomponent systems can be accurately predicted using the models trained with only 2-component data. This proves the reliability of BNN-modeled mixing rules. Thus, the apparent spectral property of the complex system is obtained by a BNN-modeled, stepwise constituent contribution protocol. Such a method is expected to compensate for the defects of traditional ML and quantum chemical calculation in predicting mixture properties. In particular, for the properties relying much on the interactions of different components of molecules, the constituent contribution protocol may provide an ideal solution with flexible modularity.

## Materials and Methods

### Data and processing

A total of 432 training sets and 40 test sets for full UV spectral prediction were collected from literature [23]. These compounds were obtained as 10 mM solutions in 100% DMSO. Each compound was diluted 50-fold (to 200 mM and 2% DMSO) with water and transferred to black, clear-bottom Greiner UV-STAR microplates. The UV absorption of each compound was read in a SpectraMax iD5 Multi-Mode Microplate spectrophotometer from 230 to 400 nm in 1-nm increments. The resulting spectra were scaled by setting the minimum absorbance to zero and normalizing to a maximum absorbance of 1.0. In order to speed up the training of the model, we increased the wavelength interval of these data to 3 nm. The processing of 3 spectral characteristics is as follows: (a) The maximum absorption wavelength is set as the wavelength at the peak of the spectrum: *λ* (as previously described, this is obviously not necessarily the maximum absorption wavelength). (b) The spectral area is the sum of absorbance at each wavelength: *S* (not the actual area, which is more conducive to model learning). (c) For some molecules without wave peak in the UV region: *H*; we set the wave peak height as the absorbance at 250 nm.

The maximum absorption wavelength data between visible light regions for comparison are obtained from literature (including 38 azo dyes dissolved in different organic solvents and 35 water-soluble rhodamine dyes). Its distribution in different solvents is shown in Table 4.[Table T4][Table T5][Table T6]

**Table 3.** Internal test results of the prediction of different methods.

Method	MAE	MSE	*R* ^2^
revGC-BNN	0.1147	0.0301	0.5417
Descriptor-BNN	0.1157	0.0298	0.6038
CGC-BNN	0.0783	0.0147	0.7782

**Table 4. T4:** Distribution of maximum absorption wavelength data in the visible light range in different solvents.

Solvent	Water	DMF	Acetone	Ethanol	Toluene	Total
**Number**	35	7	7	13	11	73

**Table 5. T5:** List of parameters of the revised group contribution method.

Conjugate descriptor	Ring		Non-ring	
	Descriptor			
*A*	N1(ortho-)	>C=	-OH	-OH(ph)
*B*	N1(meta-)	-CH=	-NH_2_	-NH_2_(ph)
	N1(para-)	-N=	-C=O(-N<)	-C=O(-N<) (ph)
	N2(α)	>N^+^=	-N<	-N<(ph)
	N2(β)	-CH_2_-	>C<	-CH_3_
	N3(α)	-HC<	-S-	-S-(ph)
	N3(β)	>C<	-O-	-O-(ph)
	N3(γ)	-O-	-F	-F(ph)
	Ar-Ar	>C=O	-Cl	-Cl(ph)
	Ar-R	-N<	-Br	-Br(ph)
		-NH-	-C=O	-OCO-(ph)
		-S-	-I	-I(ph)
		-COO-	-COOH	-COO-
		>C=C<(1)	-NO_2_	-C≡N
		>C=C<(2)	-N<(C=O)	-N<(C=O) (ph)
			-SO_2_	-SO_2_(-N<)

**Table 6. T6:** An example of input vector.

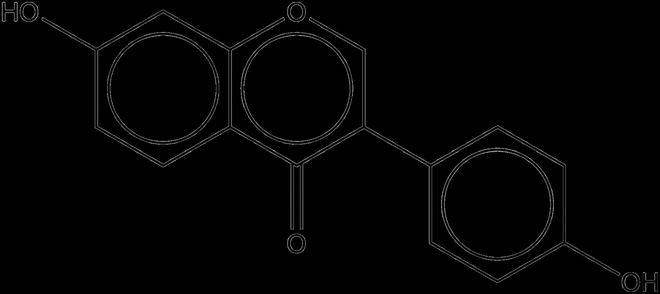				
A	B	>C=C<(1)	Ar-Ar	N2(β)
8	8	1	1	2
N1(para-)	>C=O(Ring)	-O-(Ring)	>C=(Ring)	-CH=(Ring)
1	1	1	4	8
-OH(ph)	Other groups			
2	0			

The data used for mixed spectrum prediction are obtained through experiments. We used the absorption spectra of mixed water-soluble dyes at different concentrations; and the experimental data were obtained using a CHN Spec CS-821N. The mixture spectra include the absorption spectra of single dyes and binary mixed dyes (wavelength range: 360 to 780 nm, wavelength interval: 10 nm, absorbance scale: 0 to 5). These include 36 dyes. Each dye molecule has a number in front of the SMILES format. In the data, 1.1 represents the absorption spectrum data of the dye at 0.75 × 10^−4^ mol/l, 1.2 or 1 represents the absorption spectrum data at 1 × 10^−4^mol/l, and 1.3 represents the absorption spectrum data at 1.25 × 10^−4^mol/l. The mixing data of dyes are indicated by +; 1.1 + 3.3 means that in the mixed dye solution, the concentration of 1 is 0.75 × 10^−4^mol/l, and the concentration of 3 is 1.25 × 10^−4^mol/l.

The molecular formulas of all the above data can be found in the Supplementary Materials in the format of SMILES. The complete spectral data are also stored in the Supplementary Materials in the form of absorbance at different wavelengths.

### Feature input

**Molecular and group characteris tics:** revised GC method. With this method, the structural characteristics of molecules can be converted into a computer-readable form. Some descriptors are explained as follows:

A: Total number of conjugate double bonds.

B: Maximum number of conjugated double bonds.

N1: Substitution position information on an independent aromatic ring.

N2: Number of substituents on the naphthalene ring structure. We use α, β, indicating different substitution location information.

N3: Number of substituents on aromatic rings similar to the anthracene structure. We use α, β, and γ, indicating different substitution location information.

>C=C<: 1 indicates that the aromatic ring is connected with the aromatic ring, and 2 indicates that the aromatic ring is in phase with the aliphatic ring.

In addition, in order to reduce the input characteristics as much as possible without affecting the accuracy, we do not indicate whether some groups containing double bonds, such as -COOH, are connected to the benzene ring; this information is contained in the conjugate descriptor A and B. Not only that, when C=O and -N< are connected, this structure has special effects, so it is represented separately (more details on the revised group contribution method are shown in Tables 5 and 6). Because there is a certain difference between the prediction of the UV spectrum and the visible spectrum, we used another GC method (see the Supplementary Materials for details) in the prediction of the maximum absorption wavelength in the visible light range.

**Atomic and electronic characteris tics:**

1. Electrotopological state (E-state) descriptors: This index combines the electronic states of intramolecular bonding atoms and their topological properties in the whole molecular skeleton. According to this descriptor, 3 internal states of the molecular substructure within the molecule are quantified: its element content, its valence state (electronic organization), and its topological state relative to its atomic neighbor [35].
2. Molecular operating environment (MOE-type) descriptors: The MOE-type descriptors use connectivity information and van der Waals radii to calculate the atomic van der Waals surface area contribution of an atom type to a given property, including polarizability, direct electrostatic interaction, and other factors [36].
3. Topological descriptors: According to this descriptor, the connection state of each atom is used to calculate the exponent, thus providing a highly unique exponent for a given molecule [37].
4. Connectivity descriptors.

**Solvent characteris tics:** Polarity of solvent.

In the prediction of complete spectra, we first transform molecular information into a 151-dimensional vector as input and output 3 spectral information and further merge them into a 154-dimensional vector as input and output the UV spectrum composed of a 58-dimensional vector. In the prediction of mixed spectra, we input an 86-dimensional vector composed of 2 monochromatic spectra and output a 43-dimensional mixed spectral absorption vector.

### Model and training

BNN: Deep neural network often uses gradient descent algorithm with fast convergence in realizing nonlinear regression. For small sample learning, one can sacrifice the convergence speed to obtain high accuracy. On the other hand, due to the strong learning ability of the neural network, overfitting is commonly encountered when there are few data. Therefore, we use Bayesian regularization to reduce overfitting and improve the generalization ability of the neural networks. In the Bayesian framework, the weights of the network are considered as random variables. After the data are taken, the density function for the weights can be updated according to Bayes' rule [38,39]:

$$P\left(w|D,a,\beta ,M\right)=\frac{P\left(D|w,\beta ,M\right)P\left(w|a,M\right)}{P\left(D|a,\beta ,M\right)}$$ (3)

in which *D* represents the dataset; *M* is the particular neural network model used; *w* is the vector of network weights; *P*(*w*I*α,M*) is the prior density, i.e., the known weights before any data are collected; *P*(*D*I*w,β,M*) is the likelihood function, i.e., the probability of the data occurring under the weights *w*; and *P*(*D*l*α,β,M*) is a normalization factor, which guarantees the total probability of 1 [39]. The Hessian matrix of Gauss Newton approximation can be implemented within the framework of the Levenberg–Marquardt algorithm to reduce the computing cost.

According to the established chemical characteristics, we used MATLAB to establish 3 BNN models and obtained the results by inputting the characteristics (the code and hyperparameter settings are available in the Supplementary Materials). In addition, we only divided the training set and test set in the prediction of the complete UV spectrum, the prediction of the maximum absorption wavelength of visible light was only used as a comparison for internal testing, and only 4 dyes were used in the prediction of the mixed spectrum. As mentioned above, we adopt the same form for the internal verification of all training sets: 10% of the samples are randomly selected each time as the prediction, and the average value is taken as the evaluation index after 50 repetitions. In addition, compared with 10-fold cross-validation, this method is more suitable for such a small sample. Each datum in the training set can be predicted as a prediction set for many times, and each prediction is a completely random combination. For 10-fold cross-validation, each datum has only one chance to be predicted, which may not objectively reflect the performance of the model.

We use the following indicators to evaluate the prediction performance of the model: MRE, MAE, MSE, and *R*^2^. In the prediction of complete spectrum, we will calculate the error of absorbance at each wavelength and then take the average of the errors at all wavelengths as the final error. Of course, for multiple samples, the error is the average of the errors of each sample.

## Data Availability

Data and code are available in the Supplementary Materials. It is also available at https://github.com/Fan1ing/CGC-BNN. For any questions, please contact the authors.
