# Supplementary material for: Machine Learning Spectroscopy Using a 2-Stage, Generalized Constituent Contribution Protocol
Source: Research (Wash D C). 2023 Apr 20;6:0115. doi: 10.34133/research.0115 (PMC10243197; doi:10.34133/research.0115)
Supplement: Supplementary 1 — Supplementary Text Tables S1 to S4 Figs. S1 and S2 [file research.0115.f1.zip › CGC-MC-BNN-SI_rev1.docx]

**Supporting Information**

Table of Contents

[Group contribution method 2](#_Toc120558655)

[Molecular descriptor 4](#_Toc120558656)

[Machine learning models 9](#_Toc120558657)

[Datasets 9](#_Toc120558658)

[References 10](#_Toc120558659)

# Group contribution method

In 1868, Witt proposed the hypothesis that the elements with unsaturated bonds produce color, and that the color of organic compounds is related to the chromophores in the molecule.^1^ In addition, different substituents matter for the absorption wavelength as well.^2^ However, the above qualitative cognition helps less in accurate prediction of the maximum absorption wavelength from the chemical structure due to the much too complex intermolecular and intramolecular interactions.^3^ In fact, here, each atom counts! The prediction of adsorption spectrum via quantum chemistry is based on the calculation of the vibrations of bonds, which essentially originate from the interactions between different atoms and (or) groups. This is, to some extent, analogous to the GC principle: each component as well as the interactions therein do contribute to the macroscopic properties of the system. For an accurate prediction of the adsorption spectrum, the key point is how to express the interactions between different elemental components of molecule. Thus, the GC method, which correlates thermodynamic properties quantitatively with molecular composition, came into our mind.

However, for different prediction objects, we also need to adjust the group contribution method appropriately. Here, we provide two groups division methods. For the prediction of UV spectrum, the position of substituent on aromatic ring has a great influence on the absorption of light, so we adopt the method introduced in the text. For the prediction of the maximum absorption wavelength and spectrum of visible light, the degree of conjugation of molecules has a greater impact. Therefore, in the prediction of the maximum absorption wavelength of 73 dyes, we have adopted a method to describe each atomic state as much as possible (See Table S1 for details of the method). Note that we add a conjugate descriptor C in this method, which represents the minimum number of conjugated double bonds that can be coplanar.

It should be noted that the basic principle of the two group contribution methods we use is to use the least data to achieve high accuracy. When the data is sufficient, we can combine the two methods to more accurately describe the state of groups and molecules. Not only that, for the prediction of the complete UV visible spectrum, we can respectively predict in the UV region and the visible region, and finally splice them together.

**Table S1**. List of parameters of the revGC1 method and an example of input vector

|  | Ring | |  | Non ring | |
| --- | --- | --- | --- | --- | --- |
| Descriptor | Aromatic | Non aromatic |  | -® | Non -® |
| *A* | >C= | >C= |  | -OH | -OH |
| *B* | -CH= | -CH= |  | -O- | -O- |
| *C* | -N= | -CH_2_- |  | -COO- | -COO- |
| solvent polarity | >N^+^= | -HC< |  | -N< | -N< |
|  | >C=C< | >C< |  | -NH- | -NH- |
|  |  | -N< |  | -NH_2_ | -NH_2_ |
|  |  | -NH- |  | =N^+^H_2_ | =N^+^H_2_ |
|  |  | >Si< |  | =N^+^H- | =N^+^H- |
|  |  | >P=O |  | =N^+^< | =N^+^< |
| 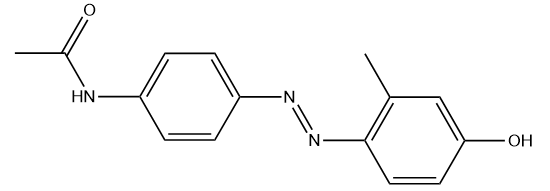 Example of the input |  | -S- |  | -S- | -S- |
| 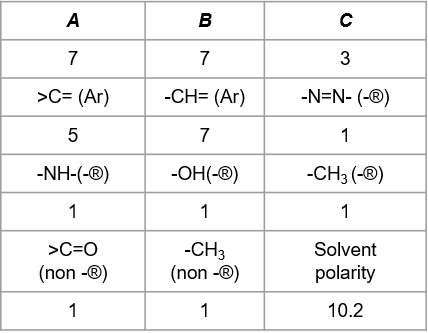 | | | | -CHO | -CHO |
|  |  |  |  | -F | -F |
|  |  |  |  | -Cl | -Cl |
|  |  |  |  | -Br | -Br |
|  |  |  |  | -SH | -SH |
|  |  |  |  | -I | -I |
|  |  |  |  | -COOH | -COOH |
|  |  |  |  | -NO_2_ | -NO_2_ |
|  |  |  |  | >C=O | >C=O |
|  |  |  |  | -N=N- | -N=N- |
|  |  |  |  | >C< | >C< |
|  |  |  |  | -C≡N | -C≡N |

# Molecular descriptor

The molecular descriptors used in this article are calculated by the RDkit toolkit. The calculation website is (<http://www.scbdd.com/chemdes/>). Table S2 shows the 92 molecular descriptors used in this paper：

**Table S2**. Molecular descriptors used in this article and their introduction.

| **Index** | **Descriptor nanme** | **Type** | **Description** | **Extended class** |
| --- | --- | --- | --- | --- |
| 1 | BalabanJ | Balaban's J index | Balaban's J value for a molecule,Chem.Phys. Lett. 89:399-404 (1982). | Topological descriptors |
| 2 | BertzCT | BertzCT | A topological index meant to quantify "complexity" of molecules.J. Am. Chem. Soc. 103:3599-601 (1981). | Topological descriptors |
| 3 | Chi0 | Chi indices | From equations (1), (9) and (10) of Rev. Comp. Chem. vol 2, 367-422, (1991) | Connectivity descriptors |
| 4 | Chi1 | Chi indices | From equations (1), (11) and (12) of Rev. Comp. Chem. vol 2, 367-422, (1991) | Connectivity descriptors |
| 5 | Chi0v | Chi indices | From equations (5), (9) and (10) of Rev. Comp. Chem. vol 2, 367-422, (1991) | Connectivity descriptors |
| 6 | Chi1v | Chi indices | From equations (5), (11) and (12) of Rev. Comp. Chem. vol 2, 367-422, (1991) | Connectivity descriptors |
| 7 | Chi2v | Chi indices | From equations (5), (15) and (16) of Rev. Comp. Chem. vol 2, 367-422, (1991) | Connectivity descriptors |
| 8 | Chi3v | Chi indices | From equations (5), (15) and (16) of Rev. Comp. Chem. vol 2, 367-422, (1991) | Connectivity descriptors |
| 9 | Chi4v | Chi indices | From equations (5), (15) and (16) of Rev. Comp. Chem. vol 2, 367-422, (1991) | Connectivity descriptors |
| 10 | Chi0n | Chi indices | Similar to Hall Kier Chi0v, but uses nVal instead of valence This makes a big difference after we get out of the first row.Rev. Comput. Chem. 2:367-422 (1991). | Connectivity descriptors |
| 11 | Chi1n | Chi indices | Similar to Hall Kier Chi1v, but uses nVal instead of valence.Rev. Comput. Chem. 2:367-422 (1991). | Connectivity descriptors |
| 12 | Chi2n | Chi indices | Similar to Hall Kier Chi2v, but uses nVal instead of valence This makes a big difference after we get out of the first row.Rev. Comput. Chem. 2:367-422 (1991). | Connectivity descriptors |
| 13 | Chi3n | Chi indices | Similar to Hall Kier Chi3v, but uses nVal instead of valence This makes a big difference after we get out of the first row.Rev. Comput. Chem. 2:367-422 (1991). | Connectivity descriptors |
| 14 | Chi4n | Chi indices | Similar to Hall Kier Chi4v, but uses nVal instead of valence.This makes a big difference after we get out of the first row.Rev. Comput. Chem. 2:367-422 (1991). | Connectivity descriptors |
| 15 | EState_VSA1 | EState_VSA | MOE-type descriptors using EState indices and surface area contributions (developed at RD, not described in the CCG paper). | E-state descriptors |
| 16 | EState_VSA2 | EState_VSA | MOE-type descriptors using EState indices and surface area contributions (developed at RD, not described in the CCG paper). | E-state descriptors |
| 17 | EState_VSA3 | EState_VSA | MOE-type descriptors using EState indices and surface area contributions (developed at RD, not described in the CCG paper). | E-state descriptors |
| 18 | EState_VSA4 | EState_VSA | MOE-type descriptors using EState indices and surface area contributions (developed at RD, not described in the CCG paper). | E-state descriptors |
| 19 | EState_VSA5 | EState_VSA | MOE-type descriptors using EState indices and surface area contributions (developed at RD, not described in the CCG paper). | E-state descriptors |
| 20 | EState_VSA6 | EState_VSA | MOE-type descriptors using EState indices and surface area contributions (developed at RD, not described in the CCG paper). | E-state descriptors |
| 21 | EState_VSA7 | EState_VSA | MOE-type descriptors using EState indices and surface area contributions (developed at RD, not described in the CCG paper). | E-state descriptors |
| 22 | EState_VSA8 | EState_VSA | MOE-type descriptors using EState indices and surface area contributions (developed at RD, not described in the CCG paper). | E-state descriptors |
| 23 | EState_VSA9 | EState_VSA | MOE-type descriptors using EState indices and surface area contributions (developed at RD, not described in the CCG paper). | E-state descriptors |
| 24 | EState_VSA10 | EState_VSA | MOE-type descriptors using EState indices and surface area contributions (developed at RD, not described in the CCG paper). | E-state descriptors |
| 25 | EState_VSA11 | EState_VSA | MOE-type descriptors using EState indices and surface area contributions (developed at RD, not described in the CCG paper). | E-state descriptors |
| 26 | FractionCSP3 | FractionCSP3 | The fraction of C atoms that are SP3 hybridized. | Constitutional descriptors |
| 27 | HallKierAlpha | HallKierAlpha | The Hall-Kier alpha value for a molecule.Rev. Comput. Chem. 2:367-422 (1991). | Topological descriptors |
| 28 | Ipc | Ipc | the information content of the coefficients of the characteristic polynomial of the adjacency matrix of a hydrogen-suppressed graph of a molecule. | Topological descriptors |
| 29 | Kappa1 | Kappa descriptors | Hall-Kier Kappa1 value | Topological descriptors |
| 30 | Kappa2 | Kappa descriptors | Hall-Kier Kappa2 value | Topological descriptors |
| 31 | Kappa3 | Kappa descriptors | Hall-Kier Kappa2 value | Topological descriptors |
| 32 | LabuteASA | LabuteASA | Labute's Approximate Surface Area (ASA from MOE) | MOE-type descriptors |
| 33 | MolMR | MolMR | Wildman-Crippen MR value.Wildman and Crippen JCICS 39:868-73 (1999) | Molecular property descriptors |
| 34 | NumAliphaticCarbocycles | NumAliphaticCarbocycles | The number of aliphatic (containing at least one non-aromatic bond) carbocycles for a molecule | Constitutional descriptors |
| 35 | NumAliphaticHeterocycles | NumAliphaticHeterocycles | The number of aliphatic (containing at least one non-aromatic bond) heterocycles for a molecule | Constitutional descriptors |
| 36 | NumAliphaticRings | NumAliphaticRings | The number of aliphatic (containing at least one non-aromatic bond) rings for a molecule | Constitutional descriptors |
| 37 | NumAromaticCarbocycles | NumAromaticCarbocycles | The number of aromatic carbocycles for a molecule | Constitutional descriptors |
| 38 | NumAromaticHeterocycles | NumAromaticHeterocycles | The number of aromatic heterocycles for a molecule | Constitutional descriptors |
| 39 | NumAromaticRings | NumAromaticRings | The number of aromatic rings for a molecule | Constitutional descriptors |
| 40 | NumHAcceptors | NumHAcceptors | Number of Hydrogen Bond Acceptors | Constitutional descriptors |
| 41 | NumHDonors | NumHDonors | Number of Hydrogen Bond Donors | Constitutional descriptors |
| 42 | NumHeteroatoms | NumHeteroatoms | Number of Heteroatoms | Constitutional descriptors |
| 43 | NumRadicalElectrons | NumRadicalElectrons | The number of radical electrons the molecule has (says nothing about spin state) | Constitutional descriptors |
| 44 | NumRotatableBonds | NumRotatableBonds | Number of Rotatable Bonds | Constitutional descriptors |
| 45 | NumSaturatedCarbocycles | NumSaturatedCarbocycles | The number of saturated carbocycles for a molecule | Constitutional descriptors |
| 46 | NumSaturatedHeterocycles | NumSaturatedHeterocycles | The number of saturated heterocycles for a molecule | Constitutional descriptors |
| 47 | NumSaturatedRings | NumSaturatedRings | The number of saturated rings for a molecule | Constitutional descriptors |
| 48 | NumValenceElectrons | NumValenceElectrons | The number of valence electrons the molecule has | Constitutional descriptors |
| 49 | PEOE_VSA1 | PEOE_VSA | MOE Charge VSA Descriptor 1 (-inf < x < -0.30) | MOE-type descriptors |
| 50 | PEOE_VSA2 | PEOE_VSA | MOE Charge VSA Descriptor 2 (-0.30 <= x < -0.25) | MOE-type descriptors |
| 51 | PEOE_VSA3 | PEOE_VSA | MOE Charge VSA Descriptor 3 (-0.25 <= x < -0.20) | MOE-type descriptors |
| 52 | PEOE_VSA4 | PEOE_VSA | MOE Charge VSA Descriptor 4 (-0.20 <= x < -0.15) | MOE-type descriptors |
| 53 | PEOE_VSA5 | PEOE_VSA | MOE Charge VSA Descriptor 5 (-0.15 <= x < -0.10) | MOE-type descriptors |
| 54 | PEOE_VSA6 | PEOE_VSA | MOE Charge VSA Descriptor 6 (-0.10 <= x < -0.05) | MOE-type descriptors |
| 55 | PEOE_VSA7 | PEOE_VSA | MOE Charge VSA Descriptor 7 (-0.05 <= x < 0.00) | MOE-type descriptors |
| 56 | PEOE_VSA8 | PEOE_VSA | MOE Charge VSA Descriptor 8 (0.00 <= x < 0.05) | MOE-type descriptors |
| 57 | PEOE_VSA9 | PEOE_VSA | MOE Charge VSA Descriptor 9 (0.05 <= x < 0.10) | MOE-type descriptors |
| 58 | PEOE_VSA10 | PEOE_VSA | MOE Charge VSA Descriptor 10 (0.10 <= x < 0.15) | MOE-type descriptors |
| 59 | PEOE_VSA11 | PEOE_VSA | MOE Charge VSA Descriptor 11 (0.15 <= x < 0.20) | MOE-type descriptors |
| 60 | PEOE_VSA12 | PEOE_VSA | MOE Charge VSA Descriptor 12 (0.20 <= x < 0.25) | MOE-type descriptors |
| 61 | PEOE_VSA13 | PEOE_VSA | MOE Charge VSA Descriptor 13 (0.25 <= x < 0.30) | MOE-type descriptors |
| 62 | PEOE_VSA14 | PEOE_VSA | MOE Charge VSA Descriptor 14 (0.30 <= x < inf) | MOE-type descriptors |
| 63 | RingCount | RingCount | The number of rings for a molecule | Constitutional descriptors |
| 64 | SMR_VSA1 | SMR_VSA | MOE MR VSA Descriptor 1 (-inf < x < 1.29) | MOE-type descriptors |
| 65 | SMR_VSA2 | SMR_VSA | MOE MR VSA Descriptor 2 (1.29 <= x < 1.82) | MOE-type descriptors |
| 66 | SMR_VSA3 | SMR_VSA | MOE MR VSA Descriptor 3 (1.82 <= x < 2.24) | MOE-type descriptors |
| 67 | SMR_VSA4 | SMR_VSA | MOE MR VSA Descriptor 4 (2.24 <= x < 2.45) | MOE-type descriptors |
| 68 | SMR_VSA5 | SMR_VSA | MOE MR VSA Descriptor 5 (2.45 <= x < 2.75) | MOE-type descriptors |
| 69 | SMR_VSA6 | SMR_VSA | MOE MR VSA Descriptor 6 (2.75 <= x < 3.05) | MOE-type descriptors |
| 70 | SMR_VSA7 | SMR_VSA | MOE MR VSA Descriptor 7 (3.05 <= x < 3.63) | MOE-type descriptors |
| 71 | SMR_VSA8 | SMR_VSA | MOE MR VSA Descriptor 8 (3.63 <= x < 3.80) | MOE-type descriptors |
| 72 | SMR_VSA9 | SMR_VSA | MOE MR VSA Descriptor 9 (3.80 <= x < 4.00) | MOE-type descriptors |
| 73 | SMR_VSA10 | SMR_VSA | MOE MR VSA Descriptor 10 (4.00 <= x < inf) | MOE-type descriptors |
| 74 | TPSA | TPSA | The polar surface area of a molecule based upon fragments | Molecular property descriptors |
| 75 | VSA_EState1 | VSA_Estate | VSA EState Descriptor 1 (-inf < x < 4.78) | E-state descriptors |
| 76 | VSA_EState2 | VSA_Estate | VSA EState Descriptor 2 (4.78 <= x < 5.00) | E-state descriptors |
| 77 | VSA_EState3 | VSA_Estate | VSA EState Descriptor 3 (5.00 <= x < 5.41) | E-state descriptors |
| 78 | VSA_EState4 | VSA_Estate | VSA EState Descriptor 4 (5.41 <= x < 5.74) | E-state descriptors |
| 79 | VSA_EState5 | VSA_Estate | VSA EState Descriptor 5 (5.74 <= x < 6.00) | E-state descriptors |
| 80 | VSA_EState6 | VSA_Estate | VSA EState Descriptor 6 (6.00 <= x < 6.07) | E-state descriptors |
| 81 | VSA_EState7 | VSA_Estate | VSA EState Descriptor 7 (6.07 <= x < 6.45) | E-state descriptors |
| 82 | VSA_EState8 | VSA_Estate | VSA EState Descriptor 8 (6.45 <= x < 7.00) | E-state descriptors |
| 83 | VSA_EState9 | VSA_Estate | VSA EState Descriptor 9 (7.00 <= x < 11.00) | E-state descriptors |
| 84 | VSA_EState10 | VSA_Estate | VSA EState Descriptor 10 (11.00 <= x < inf) | E-state descriptors |
| 85 | MaxAbsEStateIndex | Estate Index | Returns a tuple of EState indices for the molecule, Reference: Hall, Mohney and Kier. JCICS _31_ 76-81 (1991) | Topological descriptors |
| 86 | MaxAbsPartialCharge | Partial Charge | Returns molecular charge descriptors | Topological descriptors |
| 87 | MaxEStateIndex | Estate Index | Returns a tuple of EState indices for the molecule, Reference: Hall, Mohney and Kier. JCICS _31_ 76-81 (1991) | Topological descriptors |
| 88 | MaxPartialCharge | Partial Charge | Returns molecular charge descriptors | Topological descriptors |
| 89 | MinAbsEStateIndex | Estate Index | Returns a tuple of EState indices for the molecule, Reference: Hall, Mohney and Kier. JCICS _31_ 76-81 (1991) | Topological descriptors |
| 90 | MinAbsPartialCharge | Partial Charge | Returns molecular charge descriptors | Topological descriptors |
| 91 | MinEStateIndex | Estate Index | Returns a tuple of EState indices for the molecule, Reference: Hall, Mohney and Kier. JCICS _31_ 76-81 (1991) | Topological descriptors |
| 92 | MinPartialCharge | Partial Charge | Returns molecular charge descriptors | Topological descriptors |

# Machine learning models

The code we use is run in matlab R2021a on window 11. The matlab program used needs to download the neural fitting toolbox.

The parameters of the BNN models are visible in the code, and we introduced it in redme. The code output results, such as MAE, can be viewed in the variables in the matlab workspace (where T_sim1 is the average value of the prediction results).

In order to validate our models more objectively, we used several previously published machine learning models (algorithms) to predict the test set, and the results are shown in Table S3. Consequently, different machine learning models afford similar MAE and MSE upon combining our CGC method. For further comparison, we analyzed the absorption spectra fitted by different models (Including: Random forests (RF),^4^ Gradient Boosting Regression Tree (GBRT),^5^ eXtreme Gradient Boosting (Xgboost),^6^ Light Gradient Boosting Machine (LightGBM)^7^ and a deep neural network (MLP)), as shown in Figure S1. Though the spectra shapes obtained by different models are similar, the spectra afforded by BNN look more vivid.

**Table S3**. Performance of different model in the prediction of UV spectra.

| **Method** | **MAE** | **MSE** |
| --- | --- | --- |
| CGC-LightGBM | 0.0987 | 0.0232 |
| CGC-RF | 0.0948 | 0.0221 |
| CGC-GBRT | 0.0958 | 0.0227 |
| CGC-Xgboost | 0.0975 | 0.0236 |
| CGC-MLP | 0.1182 | 0.0257 |
| CGC-BNN | 0.1005 | 0.0227 |


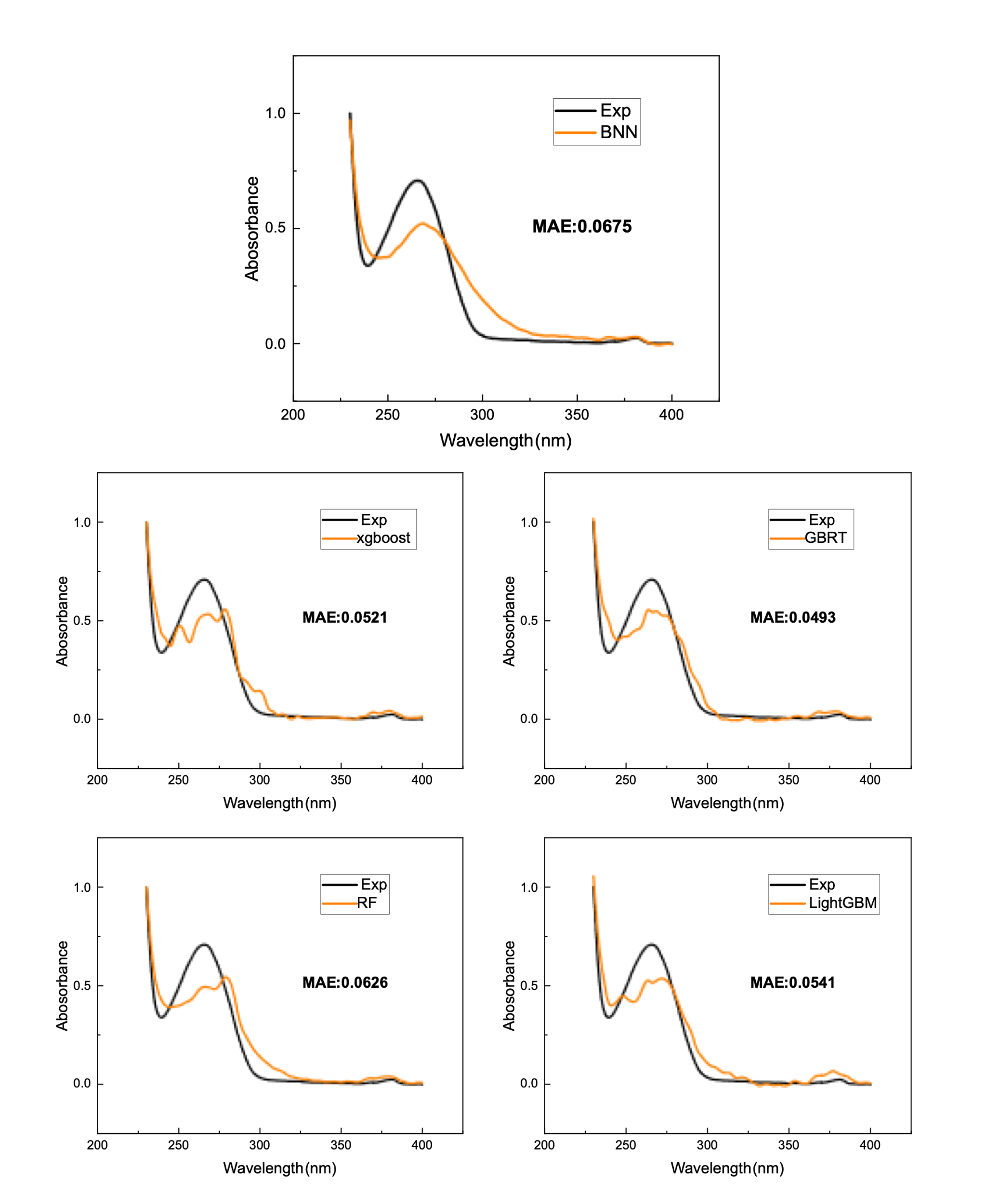


**Figure S1.** Prediction results of fluorouracil spectra by different machine learning methods.

# Molecule Contribution Method

The molecular contribution method is to solve the problem of mixture property prediction which is difficult to be solved by DFT. We intend to obtain a prediction model suitable for single component and mixture. Thus, we designed such a two-stage machine learning model and applied it to the prediction of absorption spectra. However, in the prediction of mixed spectra, when multiple components are mixed, intermolecular interaction will make the mixed spectra deviating from linear combination of single spectra. As shown in Figure S2, linear combination of single spectra of the four-component system affords large error comparing with the experimental data.


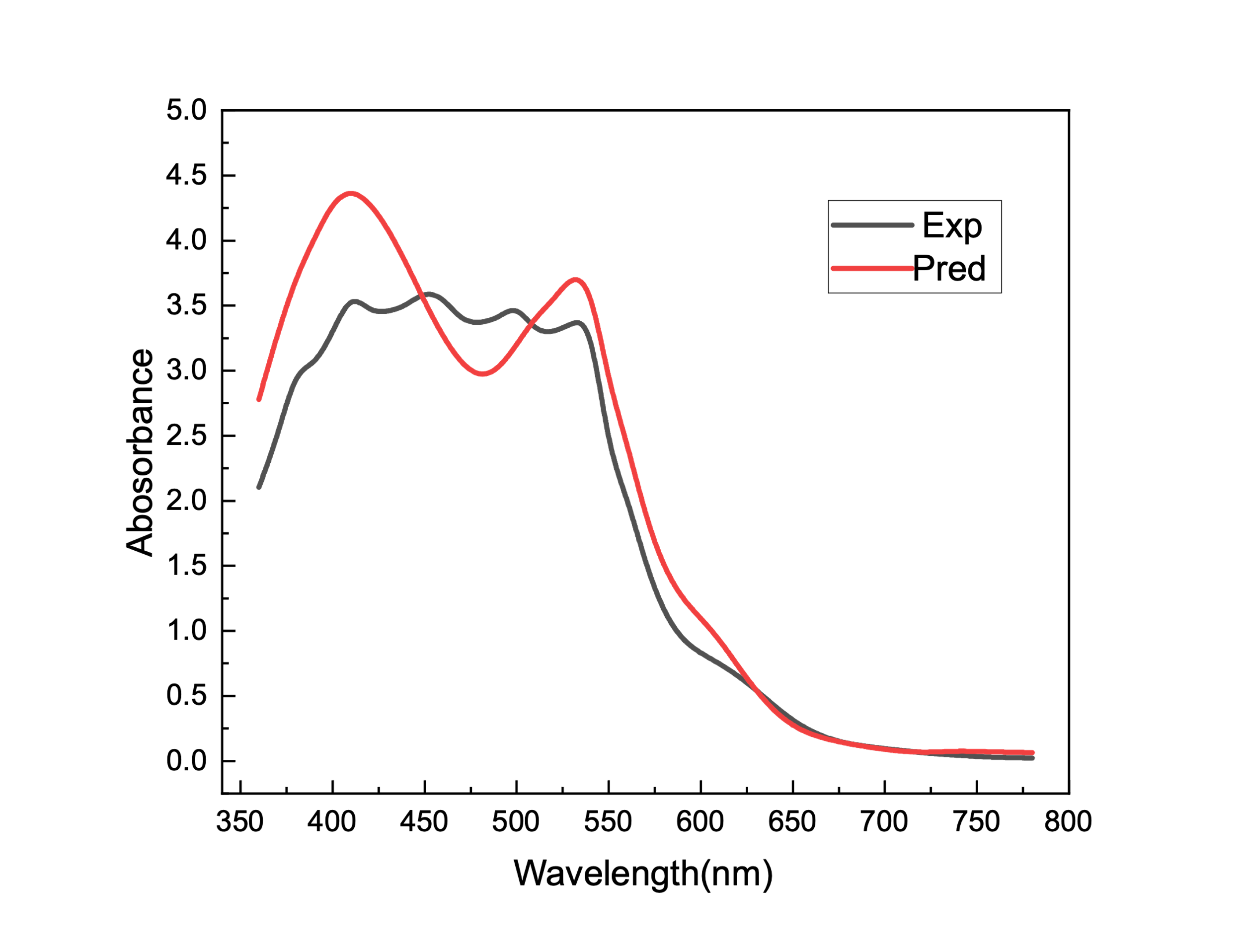


**Figure S2.** Four-component spectra obtained by linear combination of single spectra.

# Datasets

The data used in this work are from the previous reports,^8-12^ The representation of our data and the results are provided in the “**SI_mis.zip**” file, including the original codes, as listed in Table S4.

**Table S4** Explanation of the contents of excel files in the “**SI_mis.zip**” file

| In the folder “**code**” | |
| --- | --- |
| name | content |
| **Internal_validation_of_complete_UV_spectrum** | Matlab code for internal validation of complete UV spectrum. |
| **Test_of_complete_UV spectrum** | Matlab code for predicting complete uv spectrum. |
| **Test_of_three_spectral_parameters** | Matlab code for predicting three UV spectral parameters (The three parameters are represented by H,λ,S respectively. If H is predicted separately, it may not converge). |
| **internal_of _three_spectral_parameters** | Matlab code for internal validation of three UV spectral parameters. |
| **Internal_validation_of_max_abs_wavelength_of_vis** | Matlab code for internal validation of maximum absorption wavelength of visible light. |
| **MC_BNN_train** | Matlab code for internal validation of mixed spectrum. |
| **MC_BNN_TEST** | Matlab code for predicting complete mixed spectrum. |
| In the folder **“data”** | |
| **Training set of complete UV spectrum** | It contains all input characteristics and spectral experimental values. |
| **Test set of complete UV spectrum** | It contains all input characteristics and spectral experimental values. |
| **results of UV spectrum** | Prediction results of 40 test sets |
| **data of maximum absorption wavelength of visible light** | It contains all input characteristics and spectral experimental values. |
| **Raw data of mixed spectrum** | It contains all the dye structures and numbers used for mixed spectra, as well as raw data of different concentrations. |
| **reaults of mixed spectral** | Prediction results of mixed spectra. |
| **Training set of mixed spectral** | Training set for mixed spectrum prediction |

# References

1. Goodmans L. Theory and Applications of Ultraviolet Spectroscopy. *J. Am. Chem. Soc*.

2. Tatikolov AS, Krasnaya ZA, Shvedova LA, Kuzmin VA. Effects of chromophore interaction in photophysics and photochemistry of cyanine dyes. *Int. J. Photoenergy*. **2000,** *2*, 954797.

3. Shvedova, L. A.; Tatikolov, A. S. Effect of Chromophore Interactions on Spectral Properties of Ketocyanine Dyes (Review). *J. Appl. Spectrosc*. **2018,** *85* (5), 801-816.

4. Breiman, L. Random forests. *Mach. Learn.* **2001,** *45*, 5−32.

5. Friedman, J. H. Greedy function approximation: a gradient boosting machine. *Ann. Stat.* **2001,** *29*, 1189−1232.

6. Chen, T.; Guestrin, C. XGBoost: a Scalable Tree Boosting System. *arXiv*:*1603.02754v3,* **2016.**

7. Ke, G.; Meng, Q.; Finley, T.; Wang, T.; Chen, W.; Ma, W.;Ye, Q.;Liu, T. LightGBM: a highly efficient gradient boosting decision tree. NIPS’17: *Proceedings of the 31st International Conference on Neural Information Processing Systems*; Curran Associates Inc.: California, USA, New York, Dec 4–9, **2017**; pp3149–3157.

8. X, Shi.; R, Zhu.; Q, Chen. Disperse dyes containing m-cresol and their relationship between structure and color. *J. Dalian Univ. Technol.* **1988,** *S1*,151-152. (in Chinese).

9. Y, Xing.; H, Li.; L, Xu.; S, Zhi.; W, Qi.; B, Luo.; Y, Ma. Study on the relationship between structure and application properties of Azo Disperse Dyes. *Dyes and Dyeing* **2005,** *05*, 12-14+62. (in Chinese).

10. R, Lyu.; Y, Zhang.; K, Gao. Spectra and staining properties of some benzene azo-type disperse dyes. *Dye Industry* **1993,** *03*, 1-5. (in Chinese).

11. X, Chen.; K, Gao.; L, Cheng.; J, Hu. Structure and color of heterocyclic azo dyes (I.) Relationship between molecular structure and color of aminopyrimidine azo dyes. *Dye Industry*,**1986,** *03*, 18-23. (in Chinese).

12. Shao, J.; Liu, Y.; Yan, J.; Yan, Z. Y.; Wu, Y.; Ru, Z.; Liao, J. Y.; Miao, X.; Qian, L. Prediction of Maximum Absorption Wavelength Using Deep Neural Networks. *J. Chem. Inf. Model.* **2022,** *62* (6), 1368-1375.
