# Supplementary material for: Machine Learning Spectroscopy Using a 2-Stage, Generalized Constituent Contribution Protocol
Source: Research (Wash D C). 2023 Apr 20;6:0115. doi: 10.34133/research.0115 (PMC10243197; doi:10.34133/research.0115)
Supplement: Supplementary 1 — Supplementary Text Tables S1 to S4 Figs. S1 and S2 [file research.0115.f1.zip › readme.docx]

ReadMe：

1. Model introduction and structure：In order to realize the prediction from molecular structure to mixed spectrum, we have used three BNN models, which are：

（1）Spectral parameter prediction model：The model outputs three spectral parameters by inputting group contribution method and molecular descriptor (These three spectral parameters are introduced in the text). The neural network only contains a hidden layer, and the number of neurons is 6, input characteristics are normalized to 0 to 1. Other neural network parameters are: epoch=30, Training goal=0.008, Mu=0.008. (Other parameters are default values).

（2）Complete spectral prediction model: The input of this model is the input and output of the first neural network model. The neural network only contains a hidden layer, and the number of neurons is 22, input characteristics are normalized to 0 to 1. Other neural network parameters are: epoch=100, Training goal=0.008, Mu=0.008. (Other parameters are default values).

（3）Mixed spectrum prediction model: The model outputs the absorbance vector of the mixed spectrum by inputting the absorbance vector of the complete absorption spectrum (The prediction principle is shown in the figure below). The neural network only contains a hidden layer, and the number of neurons is 5, input characteristics are normalized to 0 to 1. Other neural network parameters are: epoch=30, Training goal=0.0035, Mu=0.008. (Other parameters are default values).


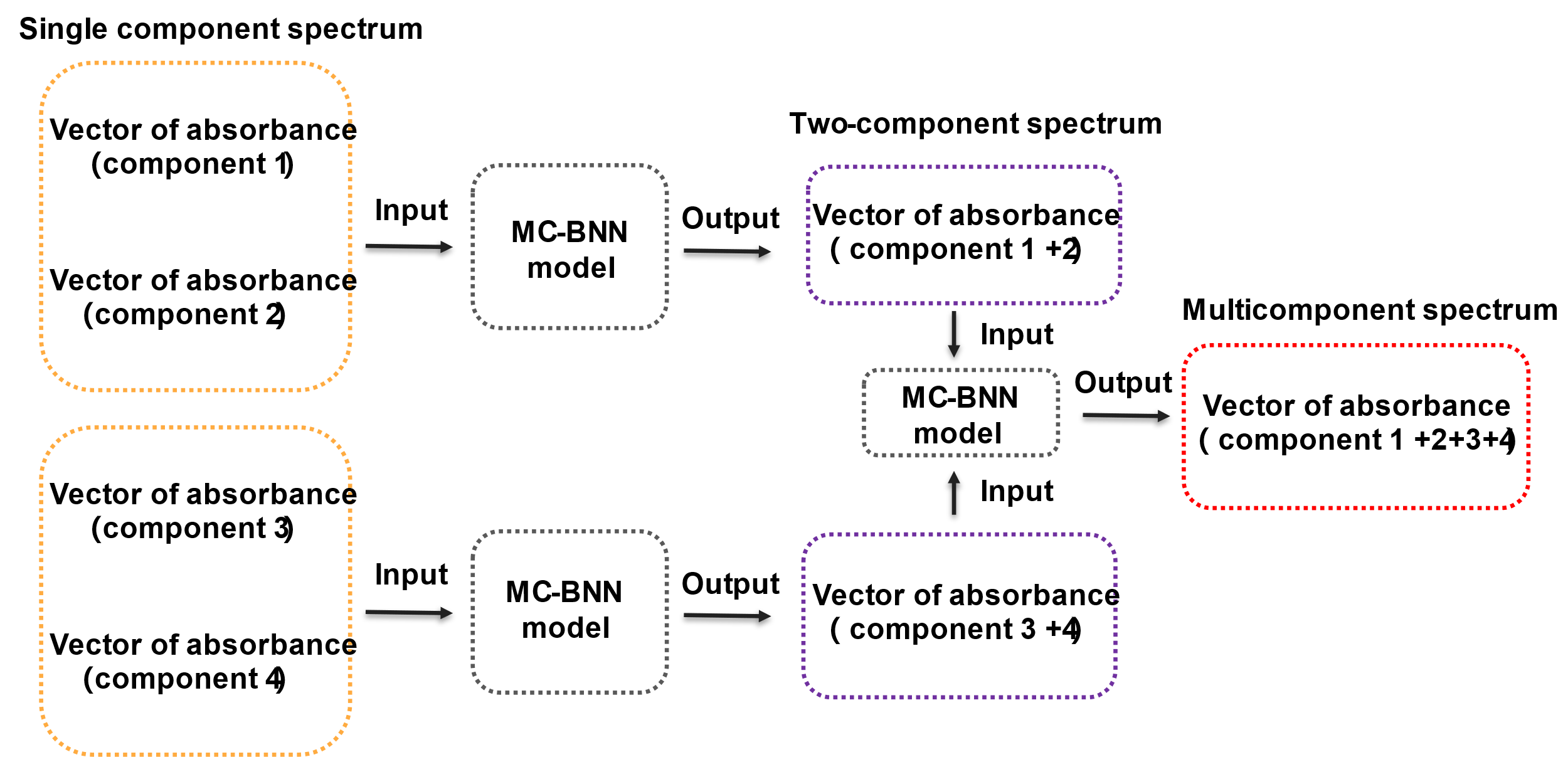


2. Code and Dataset Introduction: The following table describes the code and data set used.

| “**code**” | |
| --- | --- |
| name | content |
| **Internal_validation_of_complete_UV_spectrum** | Matlab code for internal validation of complete UV spectrum. |
| **Test_of_complete_UV spectrum** | Matlab code for predicting complete uv spectrum. |
| **Test_of_three_spectral_parameters** | Matlab code for predicting three UV spectral parameters (The three parameters are represented by H,λ,S respectively. If H is predicted separately, it may not converge). |
| **internal_of _three_spectral_parameters** | Matlab code for internal validation of three UV spectral parameters. |
| **Internal_validation_of_max_abs_wavelength_of_vis** | Matlab code for internal validation of maximum absorption wavelength of visible light. |
| **MC_BNN_train** | Matlab code for internal validation of mixed spectrum. |
| **MC_BNN_TEST** | Matlab code for predicting complete mixed spectrum. |
| **“data”** | |
| **Training set of complete UV spectrum** | It contains all input characteristics and spectral experimental values. |
| **Test set of complete UV spectrum** | It contains all input characteristics and spectral experimental values. |
| **results of UV spectrum** | Prediction results of 40 test sets |
| **data of maximum absorption wavelength of visible light** | It contains all input characteristics and spectral experimental values. |
| **Raw data of mixed spectrum** | It contains all the dye structures and numbers used for mixed spectra, as well as raw data of different concentrations. |
| **reaults of mixed spectral** | Prediction results of mixed spectra. |
| **Training set of mixed spectral** | Training set for mixed spectrum prediction |
